# Application of Mobile Virtual Reality Technology Combined with Neural Network in Facial Expression Recognition

**DOI:** 10.1155/2022/4288187

**Published:** 2022-08-05

**Authors:** Ying An

**Affiliations:** Qingdao Huanghai College, Qingdao, Shandong 266427, China

## Abstract

In recent years, in the field of virtual reality, in more and more scenes, users interact with hardware or programs through facial expressions. In order to give full play to the advantages of program interaction between virtual reality devices and users, this paper proposes a mobile virtual reality expression recognition system combined with convolution neural network. Based on the optimized AlexNet network, an expression recognition algorithm is constructed and combined with LBP feature mapping technology to improve the performance of the algorithm. At the same time, according to the nature and characteristics of mobile virtual reality devices, the user face information acquisition algorithm is optimized. The performance test results of the expression recognition system show that the recognition accuracy of the system is higher than that of the traditional convolution neural network expression recognition algorithm, and the maximum difference is greater than 10%. At the same time, the average running speed of the whole system is about 37 ms, which can meet the accuracy and real-time requirements of expression recognition in virtual reality interaction. The experimental results show that the expression recognition system proposed in this paper can be applied to mobile virtual reality devices. At the same time, it also provides new ideas for industry researchers to optimize the identification function.

## 1. Introduction

In recent years, with the continuous development of virtual reality technology, more and more people have come into view. Its core is to provide users with a sense of immersion close to reality through computer virtual environment. Now virtual reality has been widely used in military, entertainment, education, scientific research, and other fields, and it can be predicted that it will go deeper and deeper into people's daily life in the future [1]. At present, virtual reality technology mainly provides users with audiovisual upper body experience through wearing devices, and some virtual reality devices will also be attached with devices such as body sensors [2]. For mobile virtual reality devices based on head worn devices, one of the main ways to interact with users is through the user's facial expression. Therefore, an accurate and fast facial expression recognition system is necessary for mobile virtual reality devices [3]. Expression recognition is a technology that learns various facial expressions through computer algorithms, so that it can classify and judge facial expressions. It is an indispensable part of virtual reality technology and has been applied in many fields [4]. In view of this, aiming at the mobile virtual reality technology, this paper designs a set of facial expression recognition algorithm which is mainly applied to wearing mobile virtual reality devices, so as to further optimize the functions of mobile virtual reality devices and improve the user experience.

The technologies involved in this research mainly include convolutional neural network and local binary patterns (LBP) feature recognition technology. Convolutional neural network is a network structure imitating biological neural system, which can adaptively learn and classify the characteristics of the input function data. The algorithm has a good effect when it is used to recognize, classify, and judge a complex pattern. LBP technology is often used in the field of image local feature comparison. Its basic concept is to use threshold to show the difference between central point pixels and their neighborhood pixels. This technology has strong robustness to illumination. Considering that, in the use of mobile virtual reality equipment, the illumination of the user's environment is often faced with the problem of too high or insufficient intensity, therefore, introducing LBP technology into the expression recognition system of convolutional neural network can increase the recognition ability and resistance to light intensity of the recognition system.

There are two main innovations in the research. One is to optimize the traditional convolutional neural network and introduce LBP into it, which greatly optimizes the output effect of the traditional convolutional neural network. The other is to optimize the algorithm in the stage of facial region of interest recognition and pixel extraction in expression recognition, which improves the processing ability of the system in this stage to deal with the image deformation caused by user posture.

In this paper, the expression recognition system of mobile virtual reality based on convolutional neural network is analyzed. The article is divided into five parts. The first part describes and analyzes the development background of virtual reality technology. The second part describes and summarizes the current research status in the field of expression recognition and the application of convolutional neural network in various fields. The third part is the description of LBP algorithm and the optimization of neural system. The fourth part is to test the performance of the proposed system to determine whether it can be applied to mobile virtual reality devices. The last part is the summary of the full text and the elaboration of the lack of research.

## 2. Related Works

Face recognition technology has been applied in many fields, including virtual reality. Chen et al. proposed a neutral differential facial feature analysis method, which focuses on the part of the face that changes from neutral to the maximum value of facial motion when making expression. This feature has achieved constructive results through experiments based on public database, which proves its potential in the field of face recognition [5]. Li et al. constructed an integrated pruning algorithm. The main function of the algorithm is a classifier that takes into account diversity and accuracy. The researchers tested the algorithm through a variety of datasets. The evaluation results show that the effect of the algorithm is better than the current mainstream algorithm [6]. Deepak et al. proposed an expression recognition system integrating a variety of technologies and algorithms, including convolution neural network, multiangle texture processing, dense extraction, etc. The system largely eliminated the adverse effects of illumination and pose changes on expression recognition and verified the effectiveness of the system through two standard databases [7]. Considering the complexity of facial expressions and their characteristics with different meanings in different cultural backgrounds, Zia et al. designed an integrated system with incremental learning ability. The system can learn and recognize various expression patterns of expression features in different ethnic cultures. After a large number of tests, the research group believes that the system is practical [8]. Siddiqi et al. designed a face recognition model based on YouTube dataset. The model contains a new feature extraction method. Its test results show that the model achieves a weighted average recognition rate of 95% on the dataset [9].

Convolutional neural network and other models have been relatively mature applications in various fields. Rocco et al. constructed a convolutional neural network structure for set matching, which can extract and match geometric image features, detect interior points, and estimate model parameters at the same time. The test results show that the framework has good generalization ability and does not need manual annotation by the operator [10]. Fink et al. applied convolutional neural network to the detection and diagnosis of melanoma. The secondary sensitivity of the algorithm proposed by them is 88.9%, which is better than the human judgment of most dermatologists. The algorithm can well assist medical treatment [11]. Sors et al. propose a convolutional neural network system for stage scoring according to human sleep conditions. The system uses the data of sleep heart health research to train the neural network so that it can supervise and predict five human sleep stages. The test shows that its accuracy is 0.87 and has good performance and generalization [12]. Dinkla et al. applied the convolutional neural network to the magnetic resonance process of intracranial tumor radiotherapy and used the neural network to judge the required accurate dose. The experimental results showed that it only took 1 minute for each patient to calculate, and the deviation was no more than 0.02% [13]. In terms of LBP technology, Wang et al. used LBP technology to make early statistics and counting of immature green fruits to help farmers make yield prediction. This prediction is of great significance to farmers' crop management practice. The verification of this method shows that its overall accuracy reaches 85.6%, which represents its practical potential [14]. Ji et al. proposed a coding model based on texture features based on LBP, which has better accuracy and higher robustness to noise than the current model [15].

Combined with the above summary, it can be found that although there have been many researches in the field of expression recognition, there is still a lack of research for mobile virtual reality devices. Therefore, the research on expression recognition in the field of mobile virtual reality is combined with convolutional neural network with strong classification ability and LBP technology suitable for image feature extraction.

## 3. Facial Expression Recognition Algorithm for Mobile Virtual Reality Device Combined with Convolutional Neural Network

### 3.1. Face Feature Extraction Algorithm in Mobile Virtual Reality Device

**Figure 1:**
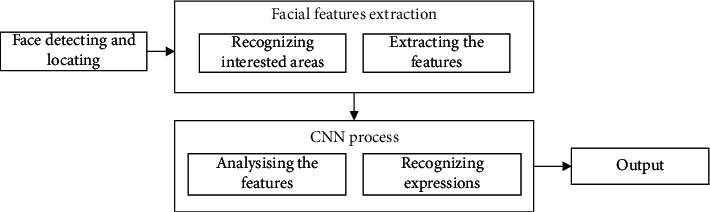
Expression recognition process of virtual reality equipment combined with convolutional neural network.

In traditional face recognition systems, whether face recognition methods based on global features or local features need to establish a good model to extract features. However, the sparse coding theory has relatively low requirements for feature selection. Therefore, combining with the feature extraction method, the sparse coding algorithm is further studied. And applied to face recognition, it expands the method and theory of face recognition and will play a certain role in promoting the research in this field, so it has very important academic significance. Recently, sparse coding SC method has made many research achievements in blind source signal separation, speech signal processing, natural image feature extraction, natural image denoising, and pattern recognition. It has important practical value and is a research hotspot in the current academic circles. Further research on sparse coding technology will not only actively promote the research of image signal processing, neural network, and other technologies, but also promote the development of new technologies in related fields. The face recognition system proposed in this study is mainly carried out according to the process shown in Figure 1. Firstly, the user's face space is located and photographed through the virtual reality device architecture and camera equipment, and then the specific interest area is extracted and the information is extracted from it. After using this information to train the convolutional neural network, the neural network can recognize the situation through this information.

In order to recognize facial expression through neural network, it is necessary to obtain the user's facial information first [16]. In this study, the way of software tracking is selected to obtain facial information. Although the accuracy of software tracking is lower than that of hardware tracking, this is a negligible factor in most application scenarios of virtual reality devices. At the same time, software tracking has the advantages of lower cost, wider universality, and adaptability to a variety of hardware devices. Vuforia image tracking technology is finally selected, which can automatically analyze the characteristics of the picture captured by the equipment, track the matching area, and provide its location information [17].

After obtaining face information, it is necessary to find out the region of interest used to recognize expression for feature extraction [18]. In the selection of the region of interest, because the change range and recognition difficulty of each part of the face in the expression change are different, and the virtual reality equipment will block or cover some areas of the face, the eyebrow eye partition and mouth partition are used as the region of interest of expression recognition. In the practical application of virtual reality devices, when users wear nonstandard or pose incorrectly, the region of interest may fail or deform [19]. In this study, perspective transformation is adopted for the region of interest to eliminate this negative impact. The principle of perspective transformation is that, in the measuring instrument, rotating the shadow forming surface or bottom surface will not affect the perspective correspondence between them. Therefore, the above problem can be improved by projecting the image operation to a new view plane by using the matrix. The expression of matrix perspective transformation is shown in formula (1).

(1) $$\begin{array}{c} \left[x\prime y\prime u\prime \right]=\left[wvu\right]\left[\begin{array}{ccc} a_{11} & a_{12} & a_{13}\\ a_{21} & a_{22} & a_{23}\\ a_{31} & a_{32} & a_{33} \end{array}\right]. \end{array}$$

In formula (1), *w* and *v* are the coordinates of the original picture, *x*, *y* are the coordinates of the picture after the perspective change, and *u* ′=*x* ′/*x*=*y* ′/*y*. In the matrix, $\left[\begin{array}{cc} a_{11} & a_{12}\\ a_{21} & a_{22} \end{array}\right]$ represents linear transformation such as scaling, $\left[\begin{array}{cc} a_{31} & a_{32} \end{array}\right]$ represents translation, and $\left[\begin{array}{c} a_{13}\\ a_{23} \end{array}\right]$ represents perspective.

The geometric meaning of the matrix transformation algorithm is to transform the region of interest of a nonpositive quadrilateral into a positive quadrilateral, and the corresponding points in the change expression can be expressed as follows,(0,0)⟶(*x*_0_, *y*_0_)，(1,0)⟶(*x*_1_, *y*_1_),(1,1)⟶(*x*_2_, *y*_2_)，(0,1)⟶(*x*_3_, *y*_3_).Then the calculation method of perspective transformation matrix can be obtained, as shown in the following equation:

(2) $$\begin{array}{c} \left[\begin{array}{ccc} a_{11} & a_{12} & a_{13}\\ a_{21} & a_{22} & a_{23}\\ a_{31} & a_{32} & a_{33} \end{array}\right]=\left[\begin{array}{ccc} x_{1}-x_{0} & y_{1}-y_{0} & \frac{\left|\begin{array}{cc} \Delta x_{3} & \Delta x_{2}\\ \Delta y_{3} & \Delta y_{2} \end{array}\right|}{\left|\begin{array}{cc} \Delta x_{1} & \Delta x_{2}\\ \Delta y_{1} & \Delta y_{2} \end{array}\right|}\\ x_{2}-x_{1} & y_{2}-y_{1} & \frac{\left|\begin{array}{cc} \Delta x_{1} & \Delta x_{3}\\ \Delta y_{1} & \Delta y_{3} \end{array}\right|}{\left|\begin{array}{cc} \Delta x_{1} & \Delta x_{2}\\ \Delta y_{1} & \Delta y_{2} \end{array}\right|}\\ x_{0} & y_{0} & a_{33} \end{array}\right],\\ \Delta x_{1}=x_{1}-x_{2},\Delta x_{2}=x_{3}-x_{2},\Delta x_{3}=x_{0}+x_{2}-x_{1}-x_{3},\Delta y_{3}=y_{0}+y_{2}-y_{1}-y_{3}=0_{{}^{\circ}}. \end{array}$$

After the perspective transformation of the region of interest is completed, the facial expression features can be extracted. Feature extraction is divided into two steps. The first step is to transform the color space of the region of interest and change the common RGB color to RG color, which can improve the efficiency and accuracy of the algorithm. The expression of color transformation is shown in

(3) $$\begin{array}{c} g=\frac{G}{\left(R+G+B\right)}.\\ r=\frac{G}{\left(R+G+B\right)}. \end{array}$$

**Figure 2:**
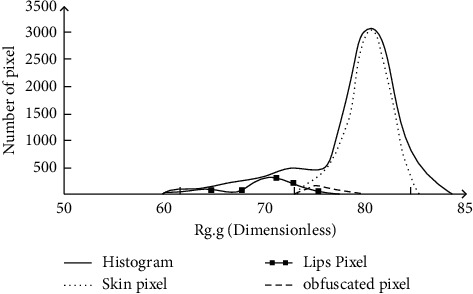
Different histogram of mouth ROI.

*R*, *G*, and *B* are the density values of three color channels under RGB color. After color conversion, the pixel distribution of the region of interest can be described in the form of color histogram.  Figure 2 is the histogram distribution of the region of interest taking lips as an example.

Figure 2 shows the pixel distribution of lips and skin in the region of interest. It can be seen that the pixel distribution of lips and skin has the characteristics of Gaussian like distribution, and the histogram description of other regions of interest also shows similar characteristics. Based on this, the feature extraction algorithm of the region of interest is designed. Firstly, the feature extraction algorithm is defined as follows, as shown in

(4) $$\begin{array}{c} th_{\text{oc}}=\frac{h_{\max}}{4},\\ th_{\text{med}}=\frac{h_{l}+h_{h}}{2}. \end{array}$$

*th*_oc_ is the ratio of the density value between the target pixel and the surrounding skin pixel in the region of interest, *th*_med_ is the median value of all columns in the histogram, *h*_max_ is the maximum value of *h*(*x*), and *h*_*l*_ and *h*_*h*_ are the first column in the histogram that is not 0 from the right and left, respectively. After confirming the definition, extract the algorithm expression, as shown in

(5) $$\begin{array}{c} h\left(x\right)>th_{oc},x>th_{\text{med}}. \end{array}$$

*h*(*x*) is the histogram distribution equation. In actual operation, the extraction can be completed by extracting all pixels that meet formula (5).

### 3.2. Optimization of Convolutional Neural Network Algorithm in Virtual Reality Environment

In this study, the improved AlexNet convolution neural network algorithm is selected to learn and judge facial expression in virtual reality environment. The main reason for choosing AlexNet convolutional neural network is that the model has faster training speed, less gradient dispersion, strong generalization ability, and less overfitting [20]. At the same time, due to the lack of diversity of the feature map generated by AlexNet model and the further improvement of the algorithm in training speed and convergence speed, a set of improvement scheme of AlexNet model is proposed.

**Figure 3:**
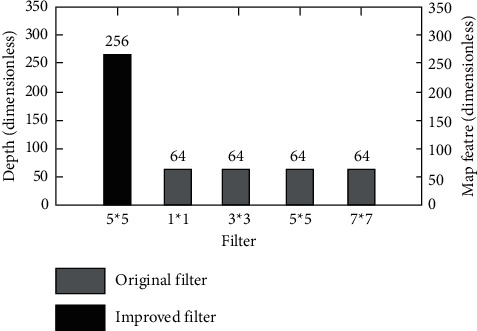
Multichannel convolution.

One of the improvements of AlexNet convolution neural network is to optimize the convolution process of the original algorithm into multichannel convolution. In the original algorithm, a convolution layer has only a single size convolution core, and multichannel convolution sets a plurality of convolution cores with different sizes in a convolution layer. Under this condition, the algorithm will generate a feature map with more feature diversity. The data comparison diagram of improved multichannel convolution and traditional convolution is shown in Figure 3.

Figure 3 shows the quantitative characteristics of multichannel convolution. It can be seen that, under the condition of maintaining the same total depth, multichannel convolution increases the feature diversity through the difference of convolution kernel size, which helps to achieve better neural network training effect.

The second improvement of AlexNet convolutional neural network is the introduction of batch normalization algorithm, which can improve the training speed of neural network on the premise of maintaining high learning rate and solve the contradiction between training speed and learning rate in neural network. The expression of data normalization in this study is shown in

(6) $$\begin{array}{c} x^{\left(k\right)}=\frac{x^{\left(k\right)}-E\left[x^{\left(k\right)}\right]}{\sqrt{\text{Var}\left[x^{\left(k\right)}\right]}}. \end{array}$$

In (6), *x*^(*k*)^ is the input training data neuron and *E*[*x*^(*k*)^] is its average value. It is found that only introducing the normalization formula will reduce the expression ability of the algorithm. In order to solve this problem, two learnable parameters are introduced into each artificial neuron, and the expression is shown in

(7) $$\begin{array}{c} y^{\left(k\right)}=\phi^{\left(k\right)}+\lambda^{\left(k\right)}y^{\left(k\right)}. \end{array}$$

*ϕ*^(*k*)^ and *λ*^(*k*)^ in formula (7) are the learnable parameters of neurons. After completing the construction of normalization expression, according to the characteristics that the calculation amount of mean and standard deviation of the algorithm is too large, an alternative calculation method is studied. This calculation method can greatly reduce the calculation amount of mean and standard deviation and optimize the algorithm. The expression of alternative mode is shown in

(8) $$\begin{array}{c} \mu =\frac{\sum_{i=1}^{m}x_{i}}{B},\sigma^{2}={\frac{\sum_{i=1}^{m}\left(x_{i}-\mu \right)}{B}}^{2}. \end{array}$$

In formula (8), *μ* is the mean, *σ* is the standard deviation, and *B* is the size of a batch. It can be seen that the calculation amount of formula (8) is much less than that of the traditional method. Finally, based on the above basis, the batch normalization expression can be obtained. The expression is shown in

(9) $$\begin{array}{c} y_{i}=\frac{\lambda \left(x_{i}-\mu \right)}{\sqrt{\sigma^{2}+\varepsilon }}+\phi . \end{array}$$

According to formula (9), the mean value and standard deviation can be obtained according to the batch size in the stage of training the neural network, but the batch size data cannot be obtained in the actual test stage. Therefore, the average value of all batch mean values completed by neural network training is used as the mean value in the test stage, and the standard deviation of each batch is estimated unbiased, and the result is used as the standard deviation of the test. So far, the batch normalization optimization of AlexNet network has been completed, and its training, convergence speed, and generalization ability have been further improved, which can better match the interactive requirements of expression recognition in virtual reality.

### 3.3. Neural Network Facial Expression Recognition Combined with LBP Feature Mapping in Virtual Reality Environment

In the actual use of virtual reality devices, it is common to cause recognition failure due to the unsatisfactory illumination of users' faces, so strategies are needed to eliminate the negative effects of illumination [21]. LBP shows good robustness to light intensity changes, and its ability to extract image local texture information is also good. Its characteristics are more suitable for assisting facial expression recognition in mobile virtual reality devices [22]. LBP is the calculated ranking value. Generally speaking, illumination is a global feature, and it is difficult to have a great impact on the pixel ranking in a small patch. Under normal circumstances, it is difficult to illuminate only one or a few pixels in an image. Therefore, LBP is not sensitive to light.

To apply LBP feature extraction, first define a coincidence function, and its expression is shown in

(10) $$\begin{array}{c} A\left(x\right)=\left\{\begin{array}{cc} 1, & x\geq 0,\\ 0, & x<0. \end{array}\right. \end{array}$$

*A* is the coincidence function, and the variable is determined by LBP process. The expression of LBP process is shown in

(11) $$\begin{array}{c} {\text{LBP}}_{P,R}=\sum_{p=0}^{p-1}2^{p}A\left(c-n\right). \end{array}$$

In formula (12), *c* is the gray value of the central point pixel of the object, and *n* is the gray value of its adjacent pixels.

However, in practical application, it is impossible to directly input the LBP processed image into the convolutional neural network for learning. The reason is that the code value of LBP is disordered, but the convolutional neural network can output meaningful results only on the premise of orderly dataset. In order to solve this problem, the multidimensional scaling method is used to input the LBP data into the convolutional neural network for learning.

When performing LBP feature mapping, first define a matrix describing the distance between all possible code values, and its expression is formula (12).

(12) $$\begin{array}{c} \Gamma ≔\left[\begin{array}{cccc} \varphi_{11} & \varphi_{12} & \cdots & \varphi_{1n}\\ \varphi_{21} & \varphi_{22} & \cdots & \varphi_{2n}\\ \vdots & \vdots & \vdots & \vdots \\ \varphi_{d1} & \varphi_{d2} & \cdots & \varphi_{dn} \end{array}\right]. \end{array}$$

In (13), *φ*_*i*,*j*_ is the distance between LBP codes *c*_*j*_ and *c*_*i*_, which represents the basic similarity of the image intensity mode corresponding to each LBP code string. In the matrix, the code value dimension is reduced by using the spatial mapping of multidimensional scaling method. The operation expression is shown in formula (13).

(13) $$\begin{array}{c} \varphi_{i,j}\approx \left\|\text{MDS}\left(c_{i}\right)-\text{MDS}\left(C_{j}\right)\right\|. \end{array}$$

*c*_*j*_ and *c*_*i*_ are object LBP codes, and MDS(*c*_*i*_) and MDS(*C*_*j*_) are their mapping values after dimensionality reduction.

During LBP operation, because the output results will eventually be applied to the facial expression recognition of mobile virtual reality devices, the research needs to explain the spatial position difference of pixel codes. Therefore, Earth mover's distance (EMD) is introduced to deal with this demand. EMD can be used to determine the difference between two LBP codes. If there are two LBP codes, the EMD expression between them is as follows:

(14) $$\begin{array}{c} \text{EMD}\left(O,P\right)=\frac{\min_{\left\{kl\right\}}\sum_{kl}f_{kl}{\text{d}}_{kl}}{\sum_{kl}f_{kl}},\sum_{kl}f_{kl}=\min\left(\sum_{k}O_{k},\sum_{l}P_{l}\right). \end{array}$$

In formula (14), ∑_*kl*_*f*_*kl*_ represents the workload required for the LBP code *O* to *P*, while *f*_*kl*_ is the workload of the LBP code from one bit to another. d_*kl*_ is the ground distance between two bit positions, which can be understood as workload in the transfer of LBP code. The definition and calculation method of d_*kl*_ are shown in

(15) $$\begin{array}{c} {\text{d}}_{kl}={\left\|k-l\right\|}_{2}. \end{array}$$

Through the EMD expression and the definition of d_*kl*_, it can be seen that EMD processing takes into account the spatial position of pixel code. So far, the research has completed the feature mapping processing of LBP, and the processed LBP image can be sent to the convolutional neural network for training.

**Figure 4:**
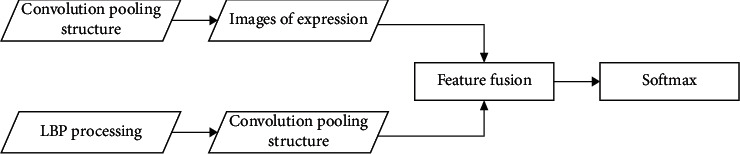
Image feature fusion.

When the neural network is trained with LBP feature mapping, the feature fusion method is adopted; that is, the LBP feature mapping map and the original map are fused during feature extraction. The advantage of this operation is that the neural network can learn the anti-illumination change characteristics of LBP and make up for some information loss that may be caused by LBP feature mapping through the original image. The schematic diagram of fusion operation flow is shown in Figure 4.

Figure 4 describes the flow of feature fusion operation. In the process, LBP image and original image are input into neural network, respectively. When they enter the convolution pool stage, respectively, they are feature fused and finally classified by softmax. So far, the research has completed the construction of neural network facial expression recognition algorithm for mobile virtual reality devices.

## 4. Performance Analysis of Expression Recognition System in Virtual Reality Environment

Since the facial expression recognition system proposed in this study is designed for mobile virtual reality devices, in order to ensure the user's interactive experience, virtual reality devices have high requirements for the processing speed, accuracy, and anti-interference ability of the expression recognition system, so the performance analysis of the system will also focus on these aspects. The experiment uses Google's board as the hardware architecture, which is widely applicable to mobile devices such as mobile phones.

**Figure 5:**
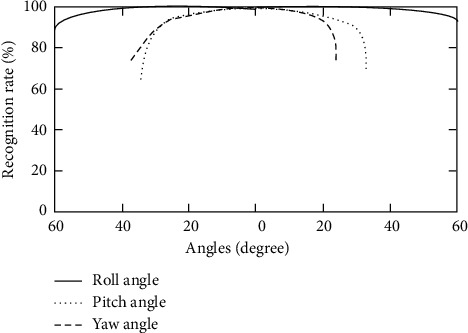
The influence of angle on facial information collecting stage.

Firstly, the algorithm of the user's face information collection and processing stage is analyzed. Under different wearing postures and angles, the recognition and pixel extraction of the user's face interest area by the recognition system are shown in Figure 5.

**Figure 6:**
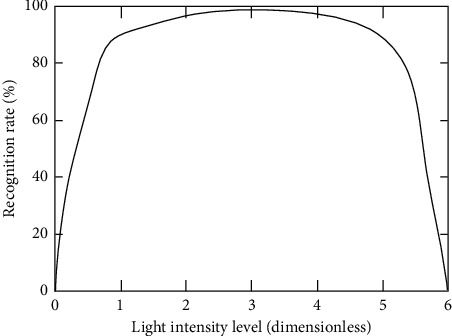
Results of feature extraction based on different light intensity.

It can be seen from Figure 5 that the virtual reality equipment can be recognized normally within the full angle range of pitch angle, yaw angle, and roll angle. Beyond this range, the recognition ability decreases rapidly to unrecognizable. Within the normal recognition range, the user's wearing posture and angle have no significant impact on the recognition effect, and the recognition effect decreases by no more than 5%. In practical application, the user can hardly notice this difference, which shows that the matrix perspective preprocessing and pixel extraction algorithm used in this study is effective. Another factor that has a great impact on the user's face information collection is the light. The control variable method is used to carry out the experiment with the light intensity as the variable on the premise of keeping other factors unchanged. The experimental results are shown in Figure 6.

Figure 6 shows the feature recognition and extraction under different light intensities. The abscissa of the image is different light intensity levels, and the ordinate is the accuracy of feature information extraction. It can be seen that the region of interest recognition and pixel extraction functions of the system are sensitive to the light intensity, and users are best to use it in an environment suitable for the light. It can be seen from the graph that when the illumination intensity is 1–5, the functions of region of interest recognition and pixel extraction of the system are relatively stable. The robustness is superior at this moment.

**Figure 7:**
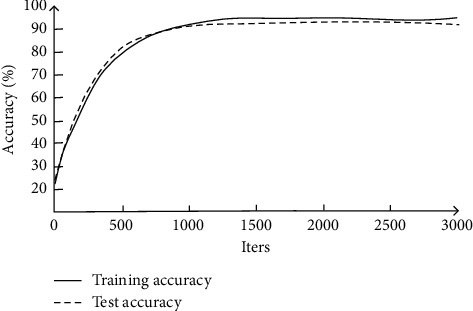
Performance of CNN expression recognition combined with LBP.

When evaluating the performance of AlexNet expression recognition algorithm combined with LBP feature mapping, we must first determine its recognition accuracy under different LBP radii, so as to find out the LBP radius with the highest recognition accuracy. After comparison, it is found that when the LBP radius is 10, the expression recognition accuracy is the highest, and its performance is shown in Figure 7.

By analyzing Figure 7, it can be found that, under the LBP radius, the neural network facial expression recognition algorithm has the fastest accuracy improvement speed between 0 and 1000 iterative steps, and the accuracy rate is about 90% at 1000 steps. After that, the slope of the curve decreases significantly and the shape is close to a straight line, indicating that the accuracy increases very slowly and approaches convergence. When the number of iteration steps reaches 3000, the accuracy of the training set converges to 91.85% and the test set converges to 91.13%. The accuracy of the algorithm under the LBP radius is stable, which is higher than other LBP radii and the convolution network without LBP. The actual accuracy is also enough to be applied to the expression recognition of virtual reality devices.

**Figure 8:**
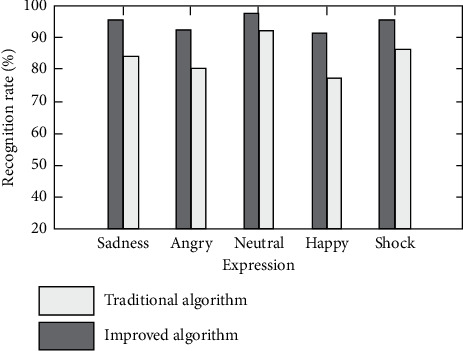
Expression recognition performance.

In the use scenario of virtual reality, the algorithm is very important for the accurate classification and recognition of different expressions, and the wrong recognition will greatly reduce the user experience. The proposed optimization algorithm divides facial expressions into anger, sadness, neutrality, joy, and surprise. The recognition of various expressions by the algorithm is shown in Figure 8.

It can be found that the recognition accuracy of the proposed algorithm for five kinds of expressions is more than 90%, and the highest neutral expression is 95.2%. The results of comparing the optimization algorithm with the traditional AlexNet neural network also show that the optimization of the algorithm is effective. Its recognition accuracy of five kinds of expressions is higher than that of the traditional algorithm, and the maximum difference is more than 13%.

**Figure 9:**
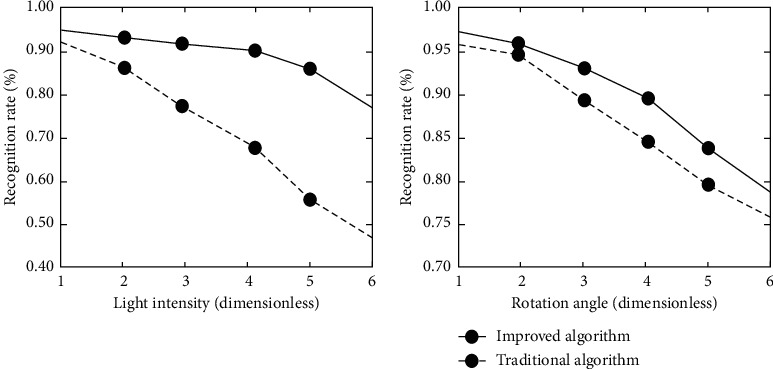
Expression recognition performance with different rotation angle and light intensity.

Due to the use posture and ambient light of virtual reality device users, the image transmitted to the recognition system is not necessarily standard, and there may be morphological changes or pixel loss. Therefore, the resistance of neural network to angle changes and light intensity changes also needs to be identified. The expression recognition rate of the algorithm under different rotation angles and illumination intensity is shown in Figure 9.

As can be seen from Figure 9, the recognition rate of traditional AlexNet neural network and optimization algorithm decreases with the increase of rotation angle, but the recognition accuracy of optimization algorithm is always higher than that of traditional algorithm. In terms of light intensity, the traditional algorithm has low resistance to light, and the recognition accuracy decreases with the change of light intensity, while the recognition accuracy of the optimization algorithm does not change much within a certain light intensity range, which shows that the neural network training effect combined with LBP is good, and the optimization algorithm has good robustness to the change of light intensity.

Before applying the proposed expression recognition system to virtual reality equipment, another project that needs to be investigated is the time-consuming operation of the system. The interaction of virtual reality environment requires the real-time response of the program to the user's expression and action. After a large number of tests, it is concluded that the average time consumption of each stage of the system is shown in Table 1.

It can be found from Table 1 that the time taken to complete a user's expression recognition is usually about 37 ms. In actual use, users will not feel the delay, which meets the requirements of virtual reality environment.

## 5. Conclusion

In recent years, virtual reality technology has the trend of lightweight and mobility. Many virtual reality software and hardware items begin to adapt to mobile devices such as mobile phones. It is one of the important interactive ways in virtual reality to interact with users through their expressions. Therefore, combined with virtual reality technology, it is necessary to use facial expression recognition algorithm for mobile devices. This research combines convolutional neural network and LBP feature mapping, proposes a facial expression recognition system for mobile virtual reality devices, and optimizes the algorithms of facial information collection and feature extraction. The performance test results of the system show that the system can normally recognize the user's facial interest areas at the full angle of pitch angle, yaw angle, and roll angle and the recognition accuracy of various expressions is stable at more than 90% and shows strong robustness to the change of light intensity. The above test data is based on the board framework with good adaptability to mobile devices such as mobile phones, so it can perform well on mobile virtual reality devices based on mobile phones and similar hardware. This research has achieved successful results, but there are still deficiencies that can be improved. The resistance of the proposed system to rotation is not much different from the traditional algorithm. At the same time, in the stage of user face information acquisition, the resistance of the system to light intensity also has room to improve, which can be used as the direction of further research. However, the resistance of the proposed system to rotation is not much different from the traditional algorithm. At the same time, in the stage of user face information collection, the resistance of the system to light intensity also has room to improve. Therefore, although this study has achieved successful results, there are still deficiencies. The research needs to be improved, so it can be used as the direction of further research.

## Figures and Tables

**Table 1:** Running time consumption.

Stage	Time
Check (ms)	0.05
Pixel processing and extracting (ms)	22.36
CNN processing (ms)	15.15
Total (ms)	37.56

## Data Availability

The data used to support the findings of this study are included within the article.
